# Supplementary material for: Feasibility and Preliminary Efficacy of American Elderberry Juice for Improving Cognition and Inflammation in Patients with Mild Cognitive Impairment
Source: Int J Mol Sci. 2024 Apr 15;25(8):4352. doi: 10.3390/ijms25084352 (PMC11050618; doi:10.3390/ijms25084352)
Supplement: Supplementary file 1 [file ijms-25-04352-s001.zip › ijms-2926997-supplementary.pdf]

Supplemental Material

Supplemental Table S1. Chemical properties of Rivers Hill elderberry juice.

Analysis	Elderberry juice
Titratable acidity (g/100 mL citric acid)	1.9±0.2
Total solids (g/100g)	9.8
Brix (°Bx)	8.3±0.1
pH	4.6±0.1
TPC (mg/L)	7624 ± 91
PAC (mg/L)	239 ± 2
Total anthocyanin content (mg/100 fresh weight)	842±80

Note. TPC: total phenolic content; PAC: proanthocyanidin content
Methods used to generate content values followed recommended procedures:
Mitch C. Johnson, Matheus Dela Libera Tres, Andrew L. Thomas, George E. Rottinghaus, and C. Michael Greenlief, "Discriminant Analyses of Anthocyanin and Polyphenol Content of American Elderberry Juice from Multiple Environments Provide Genotype Fingerprint," *Journal of Agricultural and Food Chemistry*, **65**, 4044–4050 (2017), doi.org/10.1021/acs.jafc.6b05675.

Supplemental Table S2: Quantified Average Polyphenol Content (µg/mL ± Standard Error)

	Neo-chlorogenic acid	Chlorogenic acid	Crytpto-chlorogenic acid	Quercetin 3-rutinoside	Isoquercetin	Kaempferol 3-rutinoside	Isorhamnetin3-rutinoside	Isorhamnetin 3-glucoside
**Bob Gordon Cultivar**								
(n=9)	79 ± 18	98 ± 24	4 ± 1	214 ± 90	16 ± 4	21 ± 8	72 ± 22	4.6 ± 0.5

Note. Methods used to generate content values followed recommended procedures:
Mitch C. Johnson, Matheus Dela Libera Tres, Andrew L. Thomas, George E. Rottinghaus, and C. Michael Greenlief, "Discriminant Analyses of Anthocyanin and Polyphenol Content of American Elderberry Juice from Multiple Environments Provide Genotype Fingerprint," *Journal of Agricultural and Food Chemistry*, **65**, 4044–4050 (2017), doi.org/10.1021/acs.jafc.6b05675.
